# Automated Detection of Quality Deviations in Poultry Processing Using Step-Specific YOLOv12 Models

**DOI:** 10.3390/foods15061019

**Published:** 2026-03-13

**Authors:** Daniel Einsiedel, Marco Vita, Florian Kaltenecker, Bertus Dunnewind, Johan Meulendijks, Christian Krupitzer

**Affiliations:** 1 Department of Food Informatics, University of Hohenheim, 70599 Stuttgart, Germany; daniel.einsiedel@uni-hohenheim.de (D.E.);; 2 Computational Science Hub, University of Hohenheim, 70593 Stuttgart, Germany; 3 Marel Further Processing B.V., JBT Marel, 5831 Boxmeer, The Netherlands

**Keywords:** computer vision, food processing, object detection, food quality, neural networks

## Abstract

Artificial intelligence (AI) and computer vision (CV) offer promising avenues for automated quality control in food manufacturing, yet many prior works in that sector focused on agricultural primary production tasks. This study evaluates object detection for in-line quality monitoring on a real production line for ready-to-eat chicken-type products. Overhead cameras captured images at four processing steps: forming, coating, frying, and cooking. For each step, we labeled 2000 images containing multiple products with multiple classes of quality deviations. Separate YOLOv12x models (default and hyperparameter-tuned) were trained per step and evaluated using mAP50–95, F_1_-curves, and confusion matrices. Step-specific models, i.e., models applicable solely for a specific processing step, achieved similar peak mAP50–95 (0.50–0.60), and hyperparameter tuning did not yield any major gains despite high computational cost. Performance was strongly tied to class frequency: common classes achieved high F_1_-Scores, whereas rare classes were often misclassified. To mitigate imbalance and improve robustness, we trained a single model on a combined dataset spanning all steps, which attained a higher peak mAP50–95 of 0.7331 ± 0.0040 and produced more balanced F_1_-curves, albeit with some loss of step-specific strengths, such as detection of certain deviations specific to that step. The results indicate that out-of-the-box detectors can add practical value to industrial CV-enhanced quality control in food processing, and that further improvements will primarily come from targeted data collection for minority classes, instance-centric datasets, higher-resolution or multi-scale training, and methods that address class imbalance.

## 1. Introduction

Artificial intelligence (AI) has become a major topic in recent years, with many sectors integrating it in various ways, whether by adopting existing tools or developing their own applications. The term “Artificial Intelligence” has long been familiar to the general public, thanks to popular culture. However, with the release of ChatGPT-3.5 in November 2022, it became a topic of interest to the broader public as well. Currently, tools that rely on large language models, such as ChatGPT, are among the best-known AI technologies, but they represent only one part of the broader AI landscape. Another prominent area is computer vision (CV), which has also gained momentum, as evidenced by the increasing number of model releases. For example, between 2015 and 2022, seven versions of the widely used YOLO object detectors were released, while six more have been released from 2022 to February 2025 [1,2]. AI is advancing across various fields, and while it’s not a solution to every problem, it can be a powerful tool with applications in nearly every industry. CV, for instance, is used in autonomous driving [3], medical diagnostics such as tumor detection [4], and modern industrial automation as part of Industry 4.0 [5].

Like many AI applications, CV relies heavily on machine learning, which in turn often requires large, labeled datasets. ML models can outperform humans in certain tasks; however, they usually depend on substantial human input during training, especially for data collection, preprocessing, and labeling. In the context of food, CV has been applied to a range of tasks, such as assessing the quality and safety of food products [6,7]. However, much of this work has been centered on the agricultural side of production, use cases like weed detection [8], monitoring plant health [9], or observing farm animals [10]. There is comparatively less research focused on the processing and transformation stages of raw materials into finished food products, such as meat-based convenience foods.

In this work, we use object detection, a subfield of CV, which focuses on locating and classifying objects within images. Despite the rapid evolution of “out-of-the-box” detectors such as YOLO, their deployment in industries with very specialized use-cases remains non-trivial. These environments pose significant practical challenges that are often absent in benchmark datasets. A detector might perform well in a controlled setting with even class distributions. However, industrial lines suffer from severe class imbalance, where often one class (ideally the desired one) appears more frequently than others. Furthermore, visual characteristics might appear differently at each production step. Investigating how current models handle this can be a valuable insight into the feasibility of using real-time quality assurance.

We examine multiple process steps of a real production line for ready-to-eat chicken-type products. We used images collected from cameras mounted over the production belt at multiple processing steps to train object detection models that automatically detect product quality deviations. We believe that incorporating images from a live production environment adds significant practical value. It captures natural variations, real process noise, and authentic defect appearances, making the resulting models more robust and directly applicable in practice than those trained in a controlled environment without real-world data. Additionally, by detecting not only whether a product is defective but also the type of deviation that occurs and its location, this approach enables a more detailed analysis of the underlying causes of quality issues. Based on this motivation, the following research questions guide this work:

- RQ1: Can object detection models reliably identify and localize multiple classes of quality deviations in real in-line production images across different processing steps?
- RQ2: How do step-specific object detection models compare to a unified model trained on data from all processing steps in terms of accuracy, robustness, and generalization?
- RQ3: To what extent does class imbalance, particularly rare quality deviations, affect detection performance, and which modeling or data strategies can mitigate these effects?

The work is structured as follows: Section 2 will highlight other work that applied CV in a similar context and how our work distinguishes itself from existing literature. In Section 3 we will explain our approach and methodology. The results of our work will then be presented in Section 4, and we discuss them afterward in Section 5. In Section 6 we explain potential limitations of the work. Finally, Section 7 concludes the work.

## 2. Related Works

CV has become an indispensable tool in food quality control [11]. It supports applications such as defect detection, product classification, and contamination monitoring. Earlier research relied on traditional image analysis methods and manually defined features, which were often sensitive to variations in lighting, color, or product orientation. With the advent of deep learning, convolutional neural networks (CNNs) have largely replaced conventional approaches. They enable robust feature extraction and achieve high accuracy in various food-related tasks, such as fruit quality assessment, grain classification, and surface defect detection [12,13,14]. Among deep learning models, CNNs are considered by far the most widely used architecture in food processing and inspection, distinguished by their superior feature extraction and classification capabilities [15].

The following section reviews the existing research on visual quality control. It focuses on CNN-based one-stage detectors (such as YOLO) for food products, which have established themselves as the standard for real-time object detection. In a recent study, the CNN-based model MobileNet V2 [16] was used for automated quality control and sorting of tomatoes, demonstrating its efficiency and cost-effectiveness [17]. In fish processing, various deep learning approaches have also been explored for quality assessment. For instance, one study employed the CNN-based AlexNet model [18] to automatically detect blood defects in cod fillets [19]. Another study used the Faster R-CNN model [20] to evaluate squids [21]. Additional applications in food processing include the combination of hyperspectral imaging with a 1D and 3D CNN to detect adulteration in red meat automatically [22].

The widely used YOLO model, which is also employed in this work, is increasingly being adopted in food quality control. Various versions exist most of which are provided by Ultralytics, but there are also independent repositories for some of them. Numerous studies demonstrate the successful application of YOLO in different visual food inspection tasks. One example is the automated quality assessment of tomatoes using YOLOv8 [23,24]. However, the use of YOLO for quality control in food processing environments remains limited. More frequently, YOLO is applied to food sorting tasks. For instance, YOLOv5 [25] has been used for the automated evaluation and sorting of bananas by classifying ripeness stages and detecting peel defects [26]. Another example involves the use of YOLO models for the automated detection and classification of defects in green coffee beans [27]. In a further study, YOLOv8 was applied with a focus on animal agriculture to detect livestock in complex environments [28]. A related YOLO-based application can be found in the automated quality inspection of tilapia fillets, where a YOLOv10-based model [29] was used to detect residues such as blood, scales, and bones in real time [30]. YOLO also finds widespread application in the animal-related agricultural sector, such as the use of YOLOv8 for detecting livestock in complex environments [28]. In another study, YOLO-SDD [31] was used to both detect and count farm animals in densely populated livestock housing environments [32].

YOLO is applied not only for the basic detection of livestock but also for the automated identification of pathological conditions in broiler chickens. One study successfully employed three YOLO variants (YOLOv5, YOLOv7 [33], and YOLOv8) to enable early disease detection [34]. In contrast to its widespread use in the agricultural sector, the application of YOLO in industrial food-processing environments has so far been considerably more limited. Despite this limited adoption in food-processing environments, YOLO-based approaches have already demonstrated strong performance in various other industrial manufacturing domains. For example, one study employed YOLOv5s to detect complex surface defects in metallic materials [35]. In another relevant study, AC-YOLOv5 was used for the automatic detection of textile defects [36].

In conclusion, numerous studies investigate the use of CV, particularly convolutional neural networks (CNNs), for food quality control. But the majority of these approaches are applied in the agricultural sector or to unprocessed products, whereas research in industrial food processing is significantly less common. This is noteworthy, as the more controlled conditions in industrial facilities should theoretically have a positive impact on the applicability of image recognition. Uniform lighting, spacing, and backgrounds should simplify the detection of objects and defects. The more uniform product shapes and colors of industrial foods compared to agricultural raw products should also make it easier to detect product quality deviations. Most studies also use experimental setups, whereas this study uses data from a live production, providing valuable practical insights. Compared to existing studies, there are currently no investigations that analyze the quality of processed chicken products along a production line across multiple processing stages using a YOLO model. Although related research exists in adjacent areas, such as fish processing. Furthermore, this work is distinguished by the use of the YOLOv12x-model, which represented the most recent version available at the time of the research. Consequently, only a few studies existed regarding its application in food quality control, and no known research besides our own had been conducted in the context of chicken processing. Building upon our previous work, which employed only two classes for assessing processed chicken products, this study addresses the need for a more fine-grained differentiation of quality deviations [37].

## 3. Materials and Methods

This section explains our approach, provides an overview of the investigated process line and the datasets used, and describes the models’ training and evaluation. For the training of the models, we used an NVIDIA Tesla V100 graphics card with 32 GB of VRAM on a virtual machine with 8 virtual cores (Server: AMD EPYC 7452 Dotriaconta-core (32 Core) CPU), 16 GB of RAM, using Ubuntu 22.04.5 LTS as OS and Python 3.10.12.

### 3.1. Workflow

**Figure 1:**
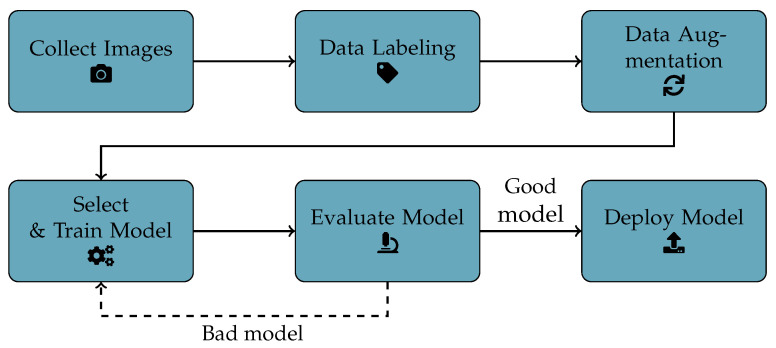
General workflow to train a CV model.

For this work, we followed a typical ML workflow for CV, which is visualized for easier understanding in Figure 1.

First, we collected images from a running production process (more details are explained in Section 3.2 and Section 3.3). The images were then labeled, which can be divided into two separate steps. The first step is the creation of bounding boxes around the objects of interest within an image. This step was automated using the tool GroundingDINO (https://github.com/IDEA-Research/GroundingDINO (accessed on 15 April 2025) [38]. GroundingDINO uses a text prompt as input and creates bounding boxes around objects. The bounding box information becomes relevant during model training, as it “tells” the model which regions are of interest, i.e., supports object detection.

In the second step, labels are attached to each bounding box, indicating what the object inside the bounding box represents. For example, if there is an image that has a dog and a cat in it, there could be two bounding boxes in the image, one with a “cat” label and one with a “dog” label. So the bounding boxes inform the model about the regions of interest, and the labels provide context about which class is present within each bounding box. This is by far the most time-consuming step of the entire process because, for a good model, a lot of high-quality data that reflects the real-world application is necessary. Additionally, the labeling needs to be consistent. When using labeling software, there is usually an option to export the data in a certain format. The YOLO models have their own format, which is a label txt file for each image, which contains the class ID and the coordinates of each bounding box in the image. During training, when an image is passed to the model, the label file provides the context of where the objects are located and their corresponding category.

As the third step, data augmentation is applied to expand the dataset by generating new data from existing data. For images, data augmentation techniques usually alter the input images in various ways. This includes transformations such as flipping or rotating the images, shuffling color channels, and adjusting brightness, to name a few [39]. This approach can aid in mitigating data imbalances by artificially increasing samples in underrepresented classes. The technique can also help the model’s generalization abilities and make it more robust to slight changes in either the data or the environment while also helping to prevent overfitting [40]. Overfitting describes a situation where the learned model becomes too specific to the training data and fails to perform effectively if the application context differs even slightly. Usually, the more recent object detection models automatically apply certain augmentations to the images while training because of these advantages. However, there are also dedicated libraries such as albumentations that can be used to apply custom augmentations to images. In our previous work [37] we experimented with custom data augmentation; however, the results did not surpass those obtained using the built-in augmentations applied during training. Therefore, in this study, we rely on the built-in augmentation pipeline. It is worth noting that data augmentation is optional; however, it is highly recommended to enlarge the dataset and increase the variability of the learned models.

Once the data is prepared, a model can be selected and trained. The selection of the YOLO12x architecture was informed by our prior comparative work [37], which indicated a performance plateau among contemporary object detection models for this specific application. Our previous work demonstrated that variations in pure detection accuracy across different state-of-the-art architectures seemed negligible. Consequently, we adopted YOLO12x to utilize the most current version of YOLO at the time of performing the study (early 2025). We specifically selected the extra-large variant despite a higher parameter count to establish a high-accuracy baseline. We chose the maximum model capacity since we wanted to determine an “upper-bound” before really considering future distillations for actual deployment.

How the training is initialized depends on the chosen model, but the general approach is always the same. First, the dataset is split into a training and a validation dataset. Commonly used percentages are 70/30 (meaning 70% of the data in the training dataset and 30% in the validation dataset), 80/20, or 90/10. Most current models can either be trained from scratch, meaning they are initialized with random weights, or fine-tuned using pre-trained weights. A pre-trained model has been trained on a large dataset (such as COCO), which can help the model converge faster and may require less data to achieve good results; however, it may also be possible that the model does not adapt perfectly to a very specific task. While there are good “out-of-the-box” solutions for object detection, there is still the option to build your own model, which might yield the best results if it is tailored to the use-case, but it also requires in-depth knowledge and skills in the field of CV. Section 3.4 describes the details of the training process applied in this study.

After training a model, several metrics can be used to evaluate it. This will be explained in more detail in Section 3.4. Based on these metrics, a decision can be made on whether the model performs well enough on the given task. If the model does not perform adequately enough, there are several options to improve the quality in an iterative process:

- Select a different model: Different models might perform better or worse on a given task.
- Change/Tune hyperparameters: Adjust certain parameters of the model, which can also be automated, but it is computationally expensive, and the time it takes depends on the used hardware.
- Collect & label more data: Collecting and labeling more data for the model to train on is currently still the option that will yield the best results. But it is also the most time-consuming and expensive option.

Once the model performs as expected, it can theoretically be deployed. After deployment, the model should be maintained and updated as needed. This last step is not within the scope of this work.

### 3.2. Process Overview

**Figure 2:**
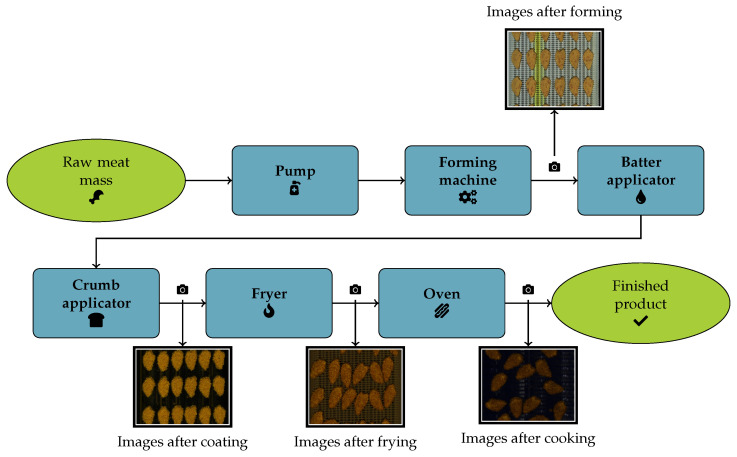
The process line investigated and used for image collection. The camera symbols (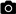) show at which positions the images where taken (adapted from [37]).

In the following, we will give an overview of the overall process from which we retrieved our data. Figure 2 shows the observed production line schematically. It is important to mention that while multiple steps are depicted and described for completeness sake, we focus on four steps in the process, where the product undergoes major changes which is also where the cameras are placed over the conveyor belt.

The product enters the process as a ground meat mass, which is pumped onto the forming machine using a vacuum pump. The forming machine consists of a drum with shaped molds, which are filled with the meat mass, which is subsequently ejected onto the production belt via air pressure. At that stage there is the first camera which collects images. The formed products then pass through a batter applicator, which applies a liquid batter to them. The batter contains spices and also acts as an adhesive for the crumb coating (cornflakes), which is applied in the next step. After the coating is applied, the next camera takes pictures of the coated products. Then the product is par-fried, which helps the coating adhere properly to the surface and adds color and flavor. Following the fryer, the third camera collects images. Last but not least is the cooking step in a spiral oven, which cooks the product to the final core temperature. After that final step, the last camera captures images of the final product, which then moves on to packaging.

### 3.3. Datasets

We collected images on three production days: 2 August 2023; 8 November 2023; and 31 January 2024 (except for the cooking step, where we only recorded on one day). To ensure high quality and consistency across the production cycle, the camera system was bolted to the line at the same distance from the belt each time. The system was furthermore equipped with a “light-shield” that should reduce interference from ambient light, within the shield a high-uniformity internal light source was used to maintain consistent illumination parameters. Images were usually taken in approx. 4-s intervals (sometimes more), which should ensure that each frame contains a unique population of products, without the risk of item duplication (no product is skipped).

For each of the four process steps, we labeled 2000 images and included 40 background images, as recommended by Ultralytics (https://docs.ultralytics.com/de/yolov5/tutorials/tips_for_best_training_results/ (accessed on 6 May 2025)). The Background images are samples containing no instances of the target objects. These serve as negative examples to teach the model to distinguish the object from environmental noise, thereby reducing false positive detections. In our case, they contain pictures of the empty production belt at each respective processing step. In each image (except the background images), approximately 20 products were labeled according to their respective classes.

While data collection occurred over multiple production shifts, the number retained from each day does vary. Since it is a real processing line, not all images were always suitable. Among other issues, things like breaks and line stoppages resulted in not useable images. For the camera of the cooking step, the hot steam of the product, plus some fat residue, obstructed the lens, which rendered the images of the first two production days useless. We tried to prioritize images where the product was properly visible, and all rows were present. The distribution of images across the distribution dates is detailed in Table 1. The dataset was split at the image-level using a randomized 80/20 split, resulting in 1632 images for training and 408 images for validation per stage.

The defect classes were defined based on repeated, structured observations during routine production. Observed deviations were grouped by characteristics and standardized into the categories presented below.

- Artifact-1: Small dark-colored specks or fragments visible on or in the surface, such as charred bits, creating visual contamination and indicating possible processing issues.
- Artifact-2: A hollow pocket or depressed area in the middle of the product caused by insufficient filling or improper forming, making the center appear thinner or sunken.
- Artifact-3: Strands of meat fibers extending beyond the intended shape of the product, giving the edge a rough, stringy, or untrimmed appearance.
- Artifact-4: Openings that pass completely through the product from one side to the other, disrupting structural integrity and potentially affecting coating adhesion or cooking performance.
- Artifact-5: Localized protrusions along the product’s perimeter, resulting in a distorted, non-uniform shape.
- Artifact-6: Areas where the breading or coating layer is incomplete or missing, leaving exposed meat and leading to inconsistent appearance and texture.
- Artifact-7: A visible gap or indentation along the outer edge of the product where meat or filling is missing, creating an irregular outline and exposing inner layers.

**Figure 3:**
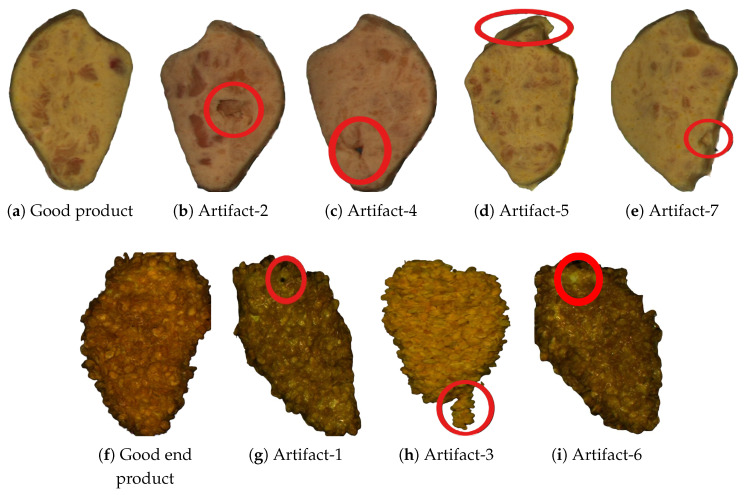
Examples of products occurring in the process. Each image represents either an observed quality deviation or products considered “good” for reference. All images in the top row were taken after the forming step, in the second row, the first image (**f**) was taken after cooking, the second and last image (**g**,**i**) after frying and the third image (**h**) after coating. (**a**) shows a properly formed product; (**b**) shows Artifact-2, missing filling at the center; (**c**) shows Artifact-4, a hole through the product, missing filling; (**d**) shows Artifact-5, an edge deformation; (**e**) shows Artifact-7, missing filling at the edge; (**f**) shows a good end product; (**g**) shows Artifact-1, dark spots on the product; (**h**) shows Artifact-3, meat fibers extending abnormally; (**i**) shows Artifact-6, missing coating on the coated product. The red circles highlight the respective artifact.

**Figure 4:**
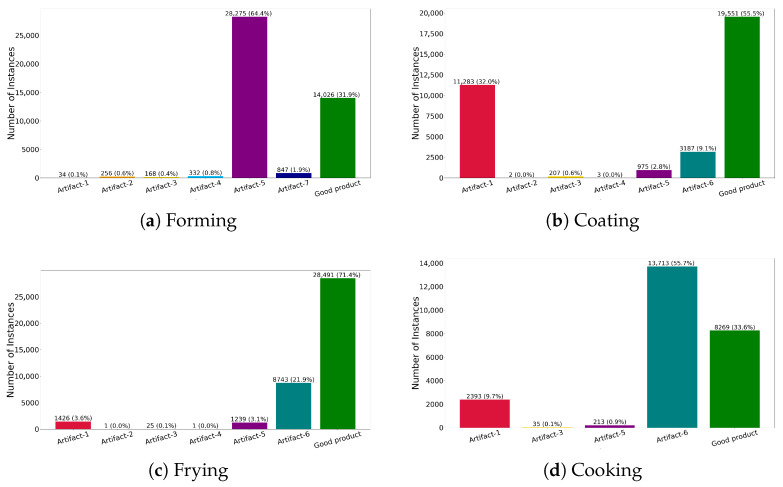
Composition of the datasets. Each graph shows the number of instances for each class that were present in the images labeled for each processing step.

It’s important to note that a product may belong to multiple classes; if so, it would receive multiple labels. In Figure 3, some images of the products are shown to give a visual example of what each of the listed items above looks like and what constitutes a “good product”. The composition of each dataset with respect to classes is shown in Figure 4.

### 3.4. Training and Evaluation

Each of the datasets mentioned above was used to train a separate YOLOv12x model (using pre-trained weights). So, for each processing step, we have a separate model tailored to that step. For the initial training, we used the default settings provided by Ultralytics (https://docs.ultralytics.com/de/models/yolo12/#usage-examples (accessed on 6 February 2026). The parameters we specified were: imgsz = 640 (all images get resized to 640 × 640 pixels), epochs = 100 (training for 100 epochs). These are default values recommended by Ultralytics, we also used these parameters in our previous work [37]. To account for variance inherent in deep learning (such as random weight initialization), we utilized a seeded training approach. For each stage, the model was trained five independent times using fixed random seeds (S = {0,1,2,3,4}). In addition to using the default models, we also conducted hyperparameter tuning for each model per processing step. We used Ultralytics’ integrated tuner class, which uses a genetic algorithm to evolve the hyperparameters after each full iteration. The epochs were kept at 100, and the iterations were set to 150. This means the model is fully trained 150 times, but each time with different hyperparameters. The search space of the tuner class is presented in Table 2.

This process is computationally intensive and costs a lot of time (e.g for the coating model we logged a runtime of 11 days, 5 h, 49 min, 42 s). We then use the best hyperparameters determined during the tuning to train the models (parameters can be found in Table A1).

A variety of metrics exist to evaluate the performance of machine learning models. Since each metric captures a different aspect of model behavior, it is useful to examine several of them. In the following, we summarize the metrics most relevant to this work, starting from the confusion matrix and its underlying terminology.

**Confusion matrix** is a table summarizing classification results using True positives (TP), False positives (FP), False negatives (FN), and True negatives (TN), helping to visualize a model’s performance across different classes [41]. For object detection with multiple classes, a single product can belong to more than one class (multi-label). Consequently, TP, FP, and FN are computed per class. The diagonal of the matrix gives us an impression on how well the model was able to detect the individual classes. The diagonal of the confusion matrix represents correct detections for each class. A value of 0 on the diagonal is not inherently negative; if a class is completely absent in the dataset, its diagonal entry is naturally zero. The “Background” class accumulates predictions that do not match any annotated class. It therefore reflects only FP and FN cases and is always zero on the diagonal. It does not correspond to the background-only images included in the dataset

**Precision** is the proportion of correctly predicted positive samples out of all predicted positives [42]:

(1) $$\mathrm{Precision}=\frac{TP}{TP+FP}$$

**Recall** is the proportion of correctly predicted positive samples out of all actual positive samples [42]:

(2) $$\mathrm{Recall}=\frac{TP}{TP+FN}$$

**F_1_-score** is the harmonic mean of precision and recall [42]:

(3) $$F_{1}=2\cdot \frac{\mathrm{Precision}\cdot \mathrm{Recall}}{\mathrm{Precision}+\mathrm{Recall}}$$

**mAP (mean Average Precision)** is the mean of the average precision values over all classes by averaging the area under the precision–recall curve for each class and then taking the mean over all classes [43]. It is often used as a general metric to compare the overall performance of different models.

**Fitness score:** The fitness score is used to determine how the model performance changes during hyperparameter tuning and is caluclated after each tuning iteration based on Equation (4).

(4) Fitnessscore=0.1·mAP50+0.9·mAP50–95

To compare how well the models handle each processing step and assess the impact of hyperparameter tuning, we used mAP (specifically mAP50–95), a standard metric for overall performance. Additionally, we inspected F_1_-scores and confusion matrices, as these provide clearer insight into the models’ capabilities, with a focus on individual classes. The mAP50–95 for the default models was aggregated across all five seeded runs for each stage and then plotted. Additionally, the peak mAP alongside the standard deviation is reported. For the confusion matrix and the F_1_-score we didn’t aggregate them across runs but chose a “representative run”. In order to do this we chose the run that was closest to the mean peak mAP of the respective stage. This should ensure that the plots represent the average performance without cherry-picking. We also evaluated the inference time relevant for the model’s viability for production environments. Since the network architecture and the input resolution remained constant across all stages, the computational complexity should remain the same across stages. We therefore used only a combined model (see Section 4.4) to represent the entire pipeline.

## 4. Results

In this section, we present the performance of YOLOv12x models trained on datasets from each processing step. First, we compare the default vs. tuned models using mAP50–95. We then examine the F_1_ scores and confusion matrices to provide a closer look at the individual classes. Last but not least, we display the results of a model trained on the combined data from all processing steps.

### 4.1. Comparison of Overall Performance and Hyperparameter Tuning

**Figure 5:**
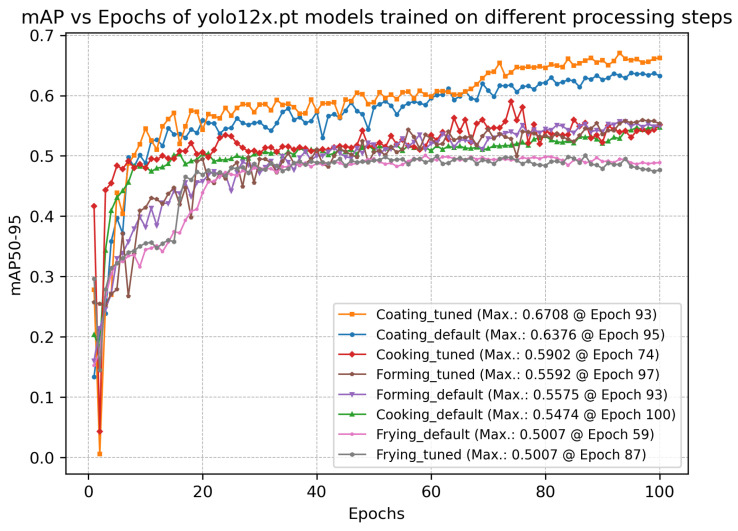
Training dynamics of YOLOv12x models across distinct processing steps. Each curve of the default models represents the epoch-wise average mAP50–95 across multiple runs using different seeds.

Figure 5 shows the overall detection performance, measured using mAP50–95. All models performed within a similar range, achieving peak mAP values around 0.50 and 0.60. The highest mAP was achieved by the tuned model trained on the coating dataset (0.6708 at epoch 93), whereas the lowest was achieved by the tuned model trained on the frying dataset (0.5007 at epoch 87). These values indicate a moderate detection performance, showing that all models were able to learn the task, but still leave room for improvement. The task does seem to be challenging for all models. Peak mAP50–95 values are listed in Table 3.

**Figure A1:**
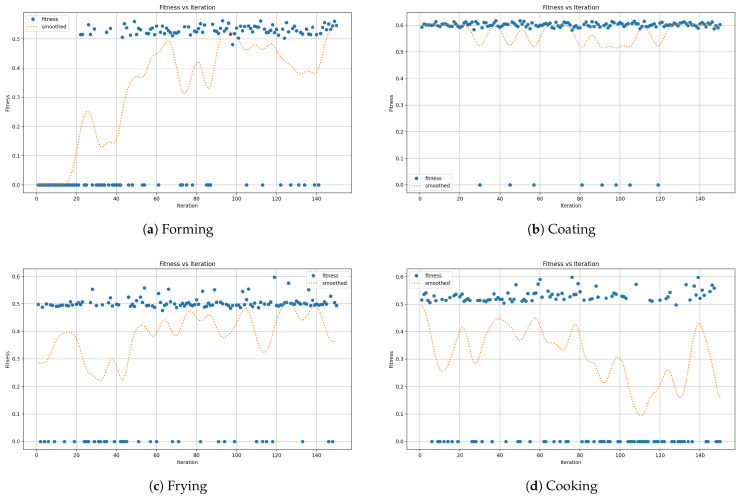
The fitness scores of the different models achieved at the different iterations of hyperparameter tuning.

The plots of the fitness scores are shown in Figure A1 and indicate whether hyperparameter tuning improved the models over time. In our case, there are no improvements in the models across iterations. These results, combined with the high computational cost of the tuning, were the main reason we decided not to pursue further hyperparameter optimization. Given the lack of observable improvement across iterations, we consider it unlikely that additional tuning would yield significant gains. For the rest of the work, we will stick to the results of the default models, as their performance is comparable to the tuned models; hence, including the latter would not provide additional meaningful insights, but they require more training time, which can be a limitation in practice.

### 4.2. F_1_-Scores

**Figure 6:**
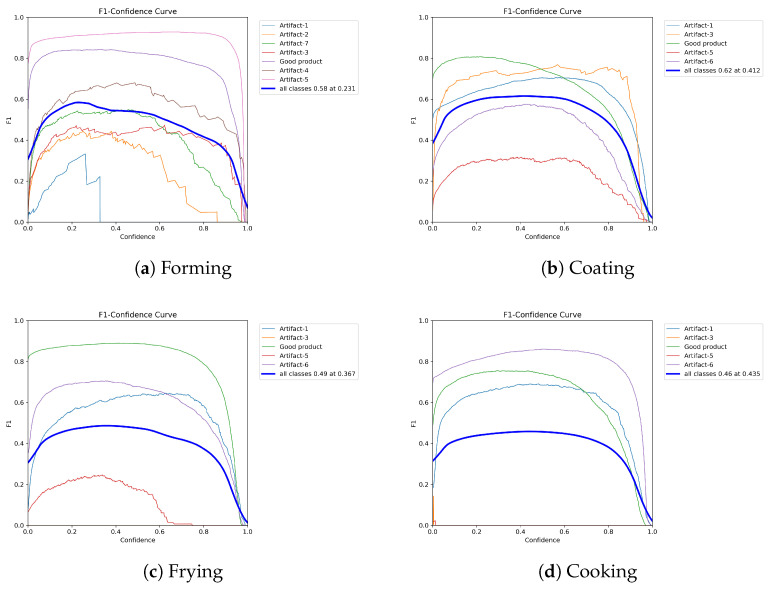
The F_1_-score of the different models, over different confidence thresholds achieved for different classes.

While the mAP gives us an overall impression of the model’s performance, it is still useful to consider other metrics that give us a more detailed insight into which classes the model learned better and which ones it struggled with. For this, we plotted the F_1_-score in Figure 6, which combines precision and recall into one metric across multiple confidence thresholds. The thick blue lines represent the overall F_1_-score across all classes. As can be seen, the models remain relatively stable across most confidence thresholds for all processing steps. In general, the classes most prevalent in each dataset achieve high F_1_ Scores and are detected and classified reliably, whereas underrepresented classes unsurprisingly yield lower scores. For example, in Figure 6a, the model after forming performs particularly well for the Artifact-5 and *Good product* classes, which are also the two most frequent in the dataset (see Figure 4a), achieving F_1_-scores of 0.8 and above. In contrast, the *Artifact-1* class, which is the least represented, barely reaches a peak F_1_-score of 0.4, and for confidence thresholds above 0.4, the score drops to zero. However, it seems that the number of instances does not seem to be the only important factor, for example, in the coating dataset, the class *Artifact-3* was detected rather well despite a low count of instances.

### 4.3. Confusion Matrices

**Figure 7:**
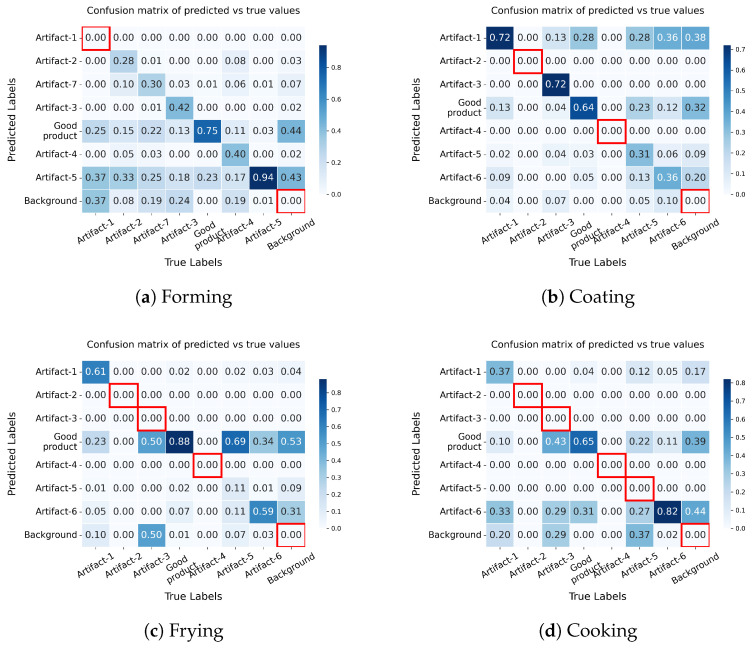
Normalized confusion matrices of the models of the different processing steps. On the x-axis, the true labels are displayed, showing the actual class, while the y-axis shows what the model predicted during validation. Zero values on the diagonal are marked in red, highlighting that these classes cannot be detected.

**Figure A2:**
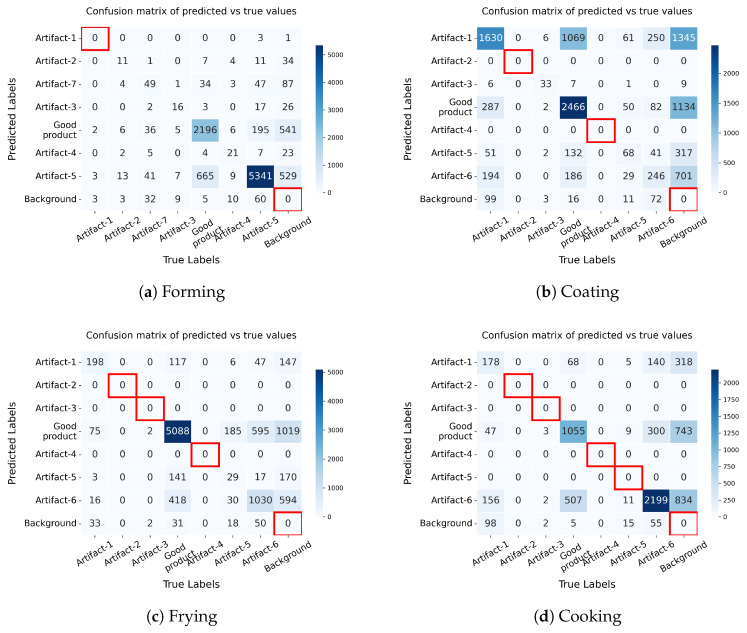
Absolute confusion matrices of the models of the different processing steps. On the x-axis the true labels are displayed, showing the actual class, while the y-axis shows what the model predicted during validation. Zero values on the diagonal are marked in red, highlighting that these classes cannot be detected.

Confusion matrices for each processing step are shown in Figure 7. These matrices provide a detailed view of the relationship between the predicted and actual classes, allowing us to identify which classes are frequently confused with one another. In this work, we present normalized confusion matrices, which allow for a direct comparison of classes regardless of their frequency in the dataset. The corresponding absolute confusion matrix, which shows the raw counts, is provided in Figure A2 in the Appendix A for completeness. In both cases, the diagonal elements represent correct predictions, with higher values indicating better classification performance. Off-diagonal values reveal misclassifications and can highlight systematic weaknesses in the model. The normalized confusion matrix for the forming step (Figure 7a) reveals notable differences in classification performance across classes.

The *Good product* class achieves the highest correct prediction rate at 75%, although it is still frequently confused with *Background* (44%). The *Artifact-5* class also performs really well with a correct rate of 94%, yet shows substantial confusion with both *Background* (43%) as well as *Artifact-1* (37%) and *Artifact-2* (33%) In contrast, classes such as *Artifact-2* and *Artifact-7* achieve correct rates of only 28% and 30%, respectively. *Artifact-3* is correctly identified in 42% of cases, with minor confusion toward *Background* (2%). Notably, *Artifact-1* are not predicted correctly at all, indicating a complete failure of the model to detect this class.

In the coating step, *Artifact-1* and *Artifact-3* achieve the highest correct prediction rates among artifacts at 72% each. The *Good product* class is correctly predicted 64% of the time, primarily suffering from confusion with *Artifact-1* (28%). Performance drops notably for *Artifact-6* and *Artifact-5*, which achieve correct rates of only 36% and 31%, respectively, and are both frequently misclassified as *Artifact-1* (36% and 28%, respectively). Additionally, background regions frequently trigger false positive detections, most notably being erroneously identified as *Artifact-1* (38%) and *Good product* (32%).

During the frying step, the model’s ability *Artifact-1* and *Artifact-6* maintain moderate correct rates of 61% and 59%, respectively. However, *Artifact-5* drops to an 11% correct rate and is predominantly misclassified as *Good product* (69%). Furthermore, *Artifact-3* is completely missed by the model; half of its instances are misclassified as *Good product* (50%), while the other half go entirely undetected as false negatives (predicted as *Background*, 50%). Finally, the background is predominantly incorrectly detected as *Good product* (53%) and *Artifact-6* (31%).

Finally, in the cooking step, *Artifact-6* emerges with the highest correct rate at 82%. The *Good product* class follows at 65%, with its primary misclassification directed toward *Artifact-6* (31%). *Artifact-1* struggles with a correct rate of only 37%, being confused with *Artifact-6* (33%) and frequently going completely undetected (20% false negatives predicted as *Background*). Similar to the frying step, the model fails entirely to detect *Artifact-3* and *Artifact-5* (both 0% correct), overwhelmingly misclassifying these actual artifacts as either *Good product* or *Artifact-6*. Moreover, background regions continue to cause false positive issues, with 44% being incorrectly identified as *Artifact-6* and 39% as *Good product*.

### 4.4. Combined Dataset

**Figure 8:**
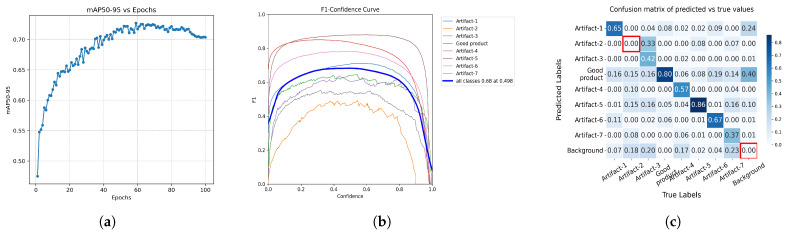
Different performance metrics of the YOLOv12x model trained on the combined dataset. (**a**) depicts the mAP50–95, (**b**) the F_1_-score for the various classes, and (**c**) shows the normalized confusion matrix. Zero values on the diagonal are marked in red, highlighting that these classes are not detected or are absent from the dataset. (**a**) Epoch-wise average mAP50–95 for the model trained on the combined dataset, aggregated across five independent runs with distinct random seeds. (**b**) F_1_ of the model trained on the combined dataset. (**c**) Normalized confusion matrix of the model trained on the combined dataset.

**Figure A3:**
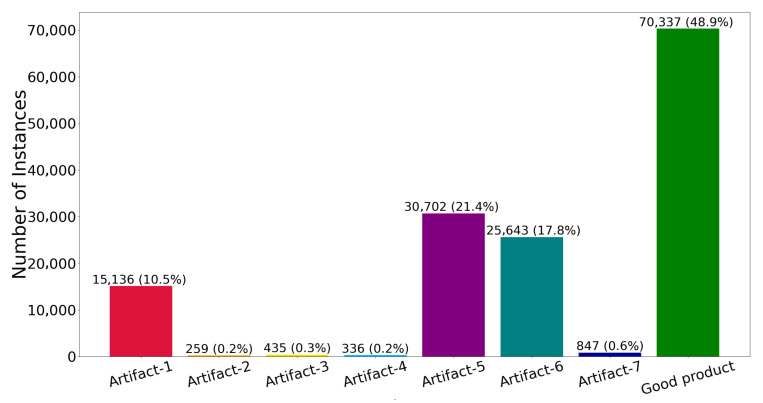
Composition of the combined dataset.

To address class imbalance and low performance for underrepresented classes, all step-specific datasets were combined into a single dataset, which we used to train another model. From our dataset it is not possible to balance out the classes, but it will combine certain minority classes across steps, leading to more instances to learn during model training. Figure A3 depicts the composition of this dataset. The resulting model achieved a mean peak mAP50–95 of 0.7331 ± 0.0040 across the seeded runs and a peak F_1_-score of 0.68 at a confidence threshold of 0.498 (Figure 8). However, there is also a trade-off: for example, the forming model (Figure 6a) achieved a higher F_1_-score for the *Artifact-5* class, and the cooking model (Figure 6d) achieved a higher F_1_-score for the *Artifact-6* class, while the coating model (Figure 6b) performed better on the *Artifact-1* and *Artifact-3*. So some of the F_1_-scores for certain classes were better in the step-specific models.

**Figure A4:**
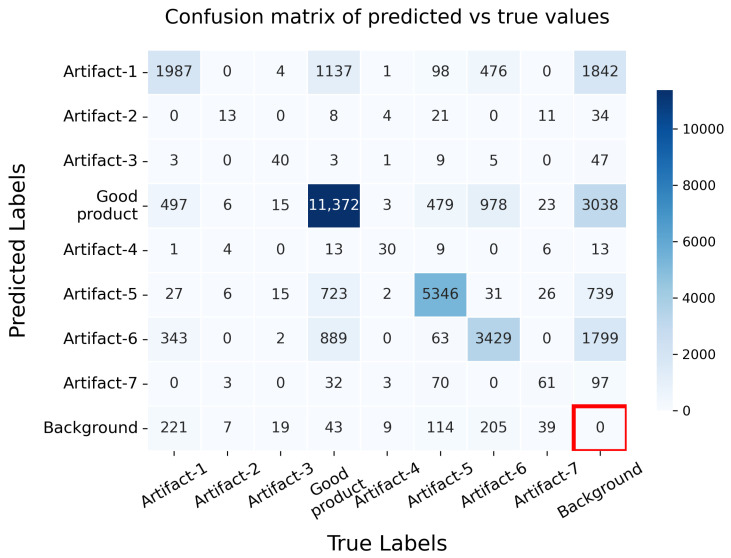
Absolute confusion matrix after validation of the YOLOv12x model trained on the combined dataset.

As can be seen in Figure 8c (the absolute confusion matrix can be found in the Appendix A, Figure A4), the combined model displays distinct performance characteristics across the classes. The *Good product* class maintains a strong correct prediction rate of 80%. Among the artifacts, *Artifact-5* achieves the highest correct rate at 86%, followed by *Artifact-6* (67%) and *Artifact-1* (65%). Other classes remain challenging; for instance, *Artifact-2*, *Artifact-3*, and *Artifact-7* achieve correct rates of only 33%, 42%, and 37%, respectively.

Crucially, background regions continue to generate false positive detections, most notably being erroneously identified as *Good product* (40%) and *Artifact-1* (24%). However, certain misclassifications improved compared to the individual models; for example, the false positive rate for *Artifact-5* (background regions incorrectly predicted as *Artifact-5*) is contained at 10%. False negative rates (actual items going undetected and being predicted as *Background*) are also present, affecting classes like *Artifact-7* (23%) and *Artifact-3* (20%) most prominently.

**Figure A5:**
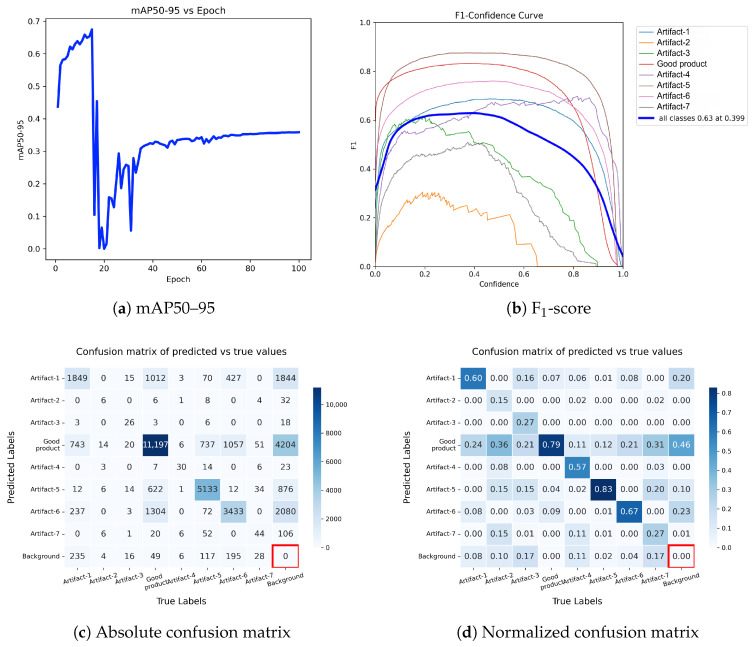
Performance metrics after training the YOLOv12x.pt model on the “best hyperparameters” as determined by 150 iterations of hyperparamter tuning using the combined dataset. (**a**) shows the mAP50–95, (**b**) depicts the F_1_-curves of each class, (**c**,**d**) show the absolute and normalized confusion matrices respectively.

**Figure A6:**
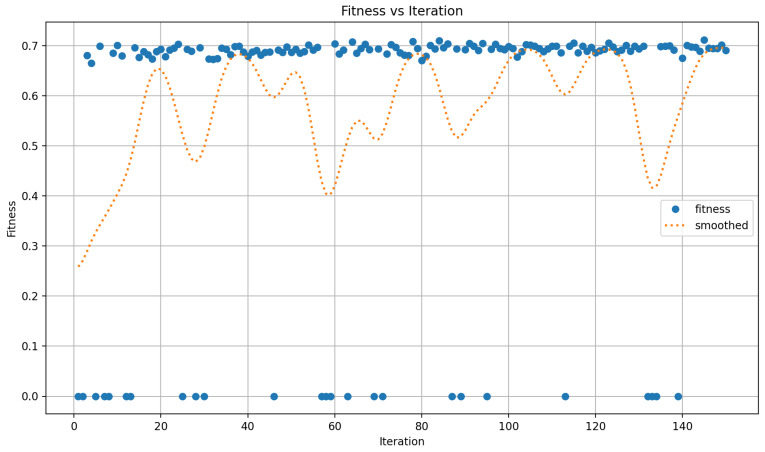
The fitness scores achieved using the combined dataset for tuning at the different iterations of hyperparameter tuning.

For completeness’s sake, we conducted a hyperparameter tuning again for this model. It took 44 days, 0 h, 1 min and 51 s but again did not yield any improvements (see Figure A5 in the Appendix A). The fitness scores achieved during tuning can also be found in the Appendix A in Figure A6.

Using the validation dataset, inference times were retrieved, which are shown in Table 4. The total processing time per image averaged 22.3 ms on our server. This latency is primarily bound by the network inference (21.0 ms), with negligible overhead from pre-processing (0.2 ms) and Non-Maximum Suppression (1.1 ms). Ultimately, this translates to a throughput of approximately 45 Frames per Second (FPS). With the belts running at around 10 m/s, this confirms the model’s capability for real-time applications. These findings are constrained by the current experimental setup. The performance metrics and computational overheads observed are specific to our existing hardware and model configurations. Practical deployment will introduce variable constraints depending on the physical architecture, specifically, whether inference is executed locally on edge devices (e.g., smart cameras) or routed to centralized compute nodes. Furthermore, processing higher-resolution images or utilizing models optimized for such scales will inherently alter the computational complexity and model accuracy, parameters that remain outside the scope of this current baseline.

## 5. Discussion

The evaluation of step-specific YOLOv12x models revealed that, despite small differences in peak mAP50–95 values, the overall detection performance was relatively similar across processing steps. Default and tuned models did not seem to differ, and hyperparameter tuning failed to yield meaningful improvements over iterations. This is not necessarily surprising, as recent state-of-the-art architectures often come with highly optimized default training strategies, meaning that further tuning might yield diminishing returns [33]. Furthermore, López de la Rosa et al. [44] demonstrated that for certain applications, data-centric factors, such as data augmentation, dataset size, variance, and image processing, play a more critical role than the choice of model or its hyperparameters.

YOLOv12x is an extra-large model with vast representational capacity, making it highly suitable for this specific task. Therefore, it is highly unlikely that performance is constrained by model underfitting; rather, the primary bottleneck appears to be data-centric. Although the dataset is representative of the production line, it inherently exhibits severe class imbalance. When the fundamental limitation is the absence of diverse, discriminative features for minority classes, optimizing hyperparameters cannot counteract this deficiency. Furthermore, as previously noted, images are downscaled to 640 × 640 pixels to manage computational load. Consequently, very small artifacts that occupy only a few pixels are downsampled even further. Hyperparameter optimization cannot recover this lost spatial data, suggesting that future improvements will likely rely on deploying higher-resolution inputs or architectures tailored for fine-grained anomaly detection. Given the absence of measurable benefits and the high computational cost, we decided to focus subsequent analyses on the default configurations.

At the class level, performance patterns were strongly associated with class frequency across the datasets. Well-represented classes, such as *Artifact-5* and *Good product* in the forming dataset, consistently achieved high F_1_-scores. In contrast, rare classes, such as *Artifact-2*, *Artifact-7*, *Artifact-1* performed less well and were in some cases not detected at all. Class imbalance is a common issue in datasets for CV, and literature (such as [45]) proposed different techniques that can help to reduce the influence on the training. This study applies (mosaic) augmentation, the technique that seemingly works best according to [45]. However, low class frequency does not necessarily always result in poor detection performance. For example, in the coating dataset, the *Artifact-3* class is far less represented than *Artifact-1* or *Good product*, yet it is detected reliably (Figure 7b). This likely reflects the presence of distinct, easily learnable features for this class.

The initial rationale to train separate models for each production step was grounded in three assumptions:

1. Step-specific specialization would enable the models to better capture the unique visual characteristics and common defect types at each stage.
2. Avoiding cross-step variation would reduce noise and limit spurious correlations.
3. Smaller datasets would shorten training times and make iterative experimentation more feasible.

While the observed results suggest that this specialization did not translate into consistently strong detection performance, they still show that, in general, it is certainly possible to apply object detection for quality control in food processing. It also demonstrates that for certain applications, out-of-the-box solutions can be implemented with minimal configuration. The comparative underperformance of the step-specific models relative to the combined model likely stems from the restricted visual variance within narrow datasets, which limits the network’s ability to extract robust, discriminative features even for its specialized task [46]. Larger datasets might be necessary, especially with less class imbalance, i.e., containing more images of the current minority classes. This, however, is quite a difficult and time-consuming endeavor. Since we took images in a running production, a lot of the products are either *Good product* or the deviations just aren’t that severe, so the likelihood of getting more of the rare classes is reduced in general. This reflects a classic distribution problem inherent to industrial visual inspection, where nominal samples often outnumber anomalous ones, making data collection for minority classes a persistent bottleneck [47].

The images contain multiple products, and certain quality deviations are quite rare. So even within an image, some classes are already underrepresented. A potential approach to solving this problem could involve extracting and labeling individual products, followed by building a dataset of individual product images per class. This approach could then be extended by using the individual class datasets to train generative augmentation methods such as generative adversarial networks (GANs), which were proposed to overcome datasets with insufficient variance [48]. By generating realistic synthetic images of rare artifacts, this method effectively reduces class imbalance prior to training the detection algorithms. Supporting this strategy, Jain et al. [49] demonstrated that employing GANs for data augmentation yields significant performance improvements in certain visual inspection tasks.

To address some limitations of step-specific models, we trained a single model on a combined dataset containing images from all processing steps. Theoretically, this approach offers several advantages: increasing the number of training examples for rare classes, exposing the model to a broader range of product appearances, and encouraging the learning of more generalizable features [46]. While this reduces the potential for step-specific optimization, it can improve robustness, particularly for underrepresented classes.

The combined model achieved a higher peak mAP50–95 (0.7320 at epoch 62) than any step-specific model and reached a peak F_1_-score of 0.68 at a confidence threshold of 0.449. Unlike the step-specific models, which often performed well for only a subset of classes, the combined model produced more balanced F_1_-score curves, with fewer instances of severe underperformance. This indicates that larger, more diverse datasets helped stabilize detection quality across categories.

Nonetheless, there were trade-offs. Some classes still performed better in their respective step-specific models. For example, *Artifact-5* in the forming model, *Artifact-6* in the cooking model, or *Artifact-1* and *Artifact-3* in the coating model retained higher peak F_1_-scores than in the combined model. Moreover, high *Background* confusion persisted for certain classes (*Good product*, *Artifact-1*, and *Artifact-6*). However, there were also clear improvements. For example, the background misclassifications for *Artifact-5* dropped from 40% in the forming model to just 10% in the combined model.

To assist practitioners in navigating these performance differences, Table 5 summarizes the core strategic trade-offs identified in our experiments. While the numerical metrics favor the unified approach for general stability, the table highlights where specialized models maintain a competitive edge.

Overall, these results suggest that while dataset combination can improve overall balance and support rare class detection, it does not guarantee universal performance gains.

However, the detection performance of these models must be interpreted within the specific operational context of the identified artifacts. Unlike safety-critical contaminants (e.g., foreign body detection), the artifacts in this study, such as uneven shapes, holes, or missing coating, affect aesthetic quality and serve as indicators of process consistency. In this specific production environment, a false positive does not necessarily result in the irreversible loss of raw materials; instead, it may simply trigger a “refeeding” mechanism that returns the product to the start of the processing line. While this introduces minor energy and throughput overhead, it avoids total product waste.

Currently, the model also operates purely on the presence and absence of a certain artifact. In a production environment, this approach might not be fine-grained enough because, for example, a hole in a product might only become an issue once it exceeds a certain surface area. Despite the moderate mAP scores, these results demonstrate that “out-of-the-box” state-of-the-art architectures can achieve functional performance levels for general quality monitoring with minimal custom engineering. In high-volume food processing, even such a moderate automated filter can significantly reduce the cognitive load on human inspectors by flagging batches with high defect densities for targeted manual review.

In general, the best options to further improve the models remain using even more labeled data and using other approaches to build more balanced datasets as described above. Especially for smaller deviations (such as Artifact-1), higher resolution might be helpful. While our uncompressed images have spatial dimensions of 1584 × 1280, they do get resized to 640 × 640 pixels during training as the computational load for training increases heavily for larger image resolutions. We tried increasing the test resolution to 1280 × 1280, but it became too computationally intensive. However, with better hardware, this would be a valid option to explore and has been shown in literature to improve model performance significantly [50]. It is important to note that this mainly targets the training phase of the models. For deployment of the models in a production setting, the computational requirements are significantly lower than during training.

## 6. Threats to Validity

Although the results of this study indicate that object detection can be applied to in-line quality control, several factors may limit the validity and generalizability of the findings. First, internal validity may be affected by inconsistencies in manual labeling, which could influence both training and evaluation outcomes. Furthermore, while hyperparameter tuning was performed, the lack of improvement might be due to an insufficient search space or not enough iterations, leaving open the possibility that better configurations exist, although we consider that unlikely.

The defect classes were defined based on structured observations during production. These definitions may not capture all relevant deviations or may differ from those used in other facilities, which limits comparability across contexts. Similarly, the chosen performance metrics (mAP and F_1_-score) provide useful insights into detection accuracy but do not fully reflect operational requirements such as inference speed, false alarm tolerance, or robustness under varying conditions.

External validity is constrained by the scope of the dataset. All images were collected from a single production line. Variations in equipment, lighting, or product types in other plants may reduce the transferability of the models, we tried to mitigate this by collecting data on different production days to include some variability. Additionally, class imbalance, particularly the underrepresentation of rare deviations, likely impacted detection performance and may not reflect the distribution of deviations in other environments.

Additionally, class imbalance, particularly the underrepresentation of rare deviations, likely impacted detection performance and may not reflect the distribution of deviations in other environments. While algorithmic mitigation strategies exist (e.g., custom focal loss, class-weighted loss functions, or complex oversampling loaders), a systematic evaluation of these methods shifts the focus from industrial application to algorithmic optimization. Modern detection architectures already incorporate highly optimized loss functions and advanced default augmentations. Furthermore, our observation that combining datasets yielded better overall performance indicates that the primary bottleneck remains dataset size and variance. Therefore, we conclude that before engineering complex sampling mechanisms, generating synthetic data, or modifying core loss functions, the most reliable and industrially safe option for deployment is to continuously collect and label more real-world data.

## 7. Conclusions

This work demonstrates that object detection can be effectively applied to in-line quality control for meat-based convenience foods using images captured from a live production environment. Across step-specific YOLOv12x models, overall performance was comparable, with peak mAP50–95 values between 0.50 and 0.60, and no visible gains from hyperparameter tuning despite substantial computational cost. Class-level analysis showed clear dependencies on class frequency: prevalent classes (e.g., *Artifact-5*, *Good product*) achieved strong F_1_-scores, while rare defects (e.g., *Artifact-1*) remained challenging and were often confused with *Background*.

To mitigate class imbalance and improve robustness, we combined all step-specific datasets into a single training set. The resulting model achieved a higher peak mAP50–95 of 0.7320 (epoch 62) and produced more balanced F_1_-score curves across classes, indicating better generalization to the diversity of product appearances encountered throughout the line. However, some step-specific strengths were diluted (e.g., detecting the deviations *Artifact-5* after forming and *Artifact-6* after cooking), and background confusion, while reduced for certain classes, persisted for others.

Overall, these results suggest that (i) out-of-the-box detectors can already add value for automated quality monitoring, (ii) data scale and class balance are the primary levers for further improvement, and (iii) specialization and generalization should be balanced according to operational priorities (e.g., maximizing overall detection vs. optimizing a few critical defects).

Future work should place a stronger emphasis on data- and task-centric improvements. A first priority is the collection of additional samples for minority defect classes, perhaps with the help of instance-level augmentation. This would ensure that rare but critical defects are detected with the same reliability as the more standard production variations.

Furthermore, the model should also be transferred to another line. This would also help apply the learned models to a completely new test set to analyze their robustness.

## Figures and Tables

**Table 1:** Distribution of images across production dates for each processing stage.

Date	Stage 1	Stage 2	Stage 3	Stage 4
2 August 2023	163	129	143	-
8 November 2023	797	1247	682	-
31 January 2024	1040	624	1176	2000
Total Labeled	2000	2000	2000	2000
Background Images	40	40	40	40
Grand Total	2040	2040	2040	2040

**Table 2:** Hyperparameter search space defined in the Ultralytics Tuner class.

Hyperparameter	Search Range (Min, Max)	Description
*Optimization*		
lr0	(1 × 10^−5^, 1 × 10^−2^)	Initial learning rate
lrf	(0.01, 1.0)	Final learning rate factor
momentum	(0.7, 0.98)	SGD momentum factor
weight_decay	(0.0, 0.001)	L2 regularization factor
warmup_epochs	(0.0, 5.0)	Number of warmup epochs
warmup_momentum	(0.0, 0.95)	Warmup initial momentum
*Loss Gains*		
box	(1.0, 20.0)	Box loss gain
cls	(0.1, 4.0)	Class loss gain
dfl	(0.4, 12.0)	Distribution Focal Loss gain
*Augmentation*		
hsv_h/s/v	(0.0, 0.1)/(0.9)/(0.9)	HSV Hue, Saturation, and Value
degrees	(0.0, 45.0)	Image rotation (+/− deg)
translate	(0.0, 0.9)	Image translation (+/− fraction)
scale	(0.0, 0.95)	Image scale (+/− gain)
shear	(0.0, 10.0)	Image shear (+/− deg)
perspective	(0.0, 0.001)	Image perspective (+/− fraction)
flipud/fliplr	(0.0, 1.0)	Flip up-down / left-right probability
bgr	(0.0, 1.0)	Channel BGR probability
mosaic	(0.0, 1.0)	Mosaic augmentation probability
mixup	(0.0, 1.0)	Mixup augmentation probability
cutmix	(0.0, 1.0)	Cutmix augmentation probability
copy_paste	(0.0, 1.0)	Segment copy-paste probability
close_mosaic	(0.0, 10.0)	Epochs to disable mosaic at the end

**Table 3:** Peak mAP50–95 for each processing step with and without hyperparameter tuning. The peaks for the default models are mean values from the different seeded runs independent from epoch.

Processing Step	Peak mAP50–95	Epoch
Forming	0.5689 ± 0.0073	-
Forming (tuned)	0.5592	97
Coating	0.6405 ± 0.0253	-
Coating (tuned)	0.6708	91
Frying	0.5041 ± 0.0021	-
Frying (tuned)	0.5007	87
Cooking	0.5549 ± 0.0220	-
Cooking (tuned)	0.5902	74

**Table 4:** Detailed breakdown of YOLO12x inference latency and throughput across the validation set. Tests were conducted at a 640 × 640 resolution with a batch size of 1.

Metric/Component	Value
Validation Set Size	1628 images
Image Pre-processing	0.2 ms/image
Network Inference	21.0 ms/image
NMS Post-processing	1.1 ms/image
Total Latency	22.3 ms/image
Throughput	∼45 FPS
Hardware	NVIDIA Tesla V100 32 GB VRAM

**Table 5:** Strategic Trade-offs: Unified vs. Step-Specific Modeling for Industrial Quality Assurance.

Dimension	Unified Model (All Steps)	Step-Specific Models (4×)
Overall Accuracy	Higher baseline mAP due to feature sharing and larger dataset diversity.	Variable; highly accurate for stage-specific textures but lacks global context.
Critical Defects	At times lower sensitivity to niche deviations.	Better for stage-specific defects.
Data Strategy	**Centralized:** All images pooled into one master set.	**Segmented:** Images are sorted and labeled according to stage.
Deployment	**Low Complexity:** Single model instance; lower GPU/CPU overhead.	**High Complexity:** Multiple models; higher hardware requirements.
Robustness	Better generalization to global factory changes (e.g., lighting, lens blur).	High robustness to “process noise” specific to a single station.
Scalability	High; new data from any stage improves the shared model.	Low; adding a new production step requires a dedicated training cycle and deployment.

## Data Availability

The datasets presented in this article are not readily available because the datasets due to being obtained at a third party facility. Requests to access the datasets should be directed to the corresponding author.

## Abbreviations

The following abbreviations are used in this manuscript:

AI	Artificial Intelligence
CNN	Convolutional Neural Network
COCO	Common Objects in Context
CPU	Central Processing Unit
CV	Computer Vision
FN	False Negative
FP	False Positive
ML	Machine Learning
mAP	Mean Average Precision
OS	Operating System
RAM	Random Access Memory
R-CNN	Region-based Convolutional Neural Network
TN	True Negative
TP	True Positive
VRAM	Video RAM
YAML	YAML Ain’t Markup Language
YOLO	You Only Look Once

## Appendix A

**Table A1:** List of the default hyperparameters and the “best” hyperparameters obtained after tuning.

Parameter	Default	Forming	Coating	Frying	Cooking	Combined Dataset
lr0	0.01000	0.00683	0.00812	0.00848	0.01134	0.00675
lrf	0.01000	0.00593	0.00615	0.01597	0.00929	0.01164
momentum	0.93700	0.94229	0.83517	0.75489	0.88614	0.92901
weight_decay	0.00050	0.00089	0.00050	0.00074	0.00060	0.00050
warmup_epochs	3.00000	2.75271	2.34845	3.50775	4.21883	2.87595
warmup_momentum	0.80000	0.42819	0.65240	0.48878	0.91129	0.76609
warmup_bias_lr	0.10000	0.10000	0.10000	0.10000	0.10000	0.10000
box	7.50000	5.44443	8.26254	7.68924	6.35630	8.00000
cls	0.50000	0.24015	0.30007	0.35557	0.33828	0.25996
dfl	1.50000	0.90330	1.40660	1.32605	1.72579	1.35213
pose	12.00000	12.00000	12.00000	12.00000	12.00000	12.00000
kobj	1.00000	1.00000	1.00000	1.00000	1.00000	1.00000
nbs	64	64	64	64	64	64
hsv_h	0.01500	0.01210	0.01360	0.01378	0.02181	0.00953
hsv_s	0.70000	0.67892	0.55069	0.61348	0.42688	0.63210
hsv_v	0.40000	0.44494	0.32865	0.46135	0.62898	0.33176
degrees	0.00000	0.00000	0.00000	0.00000	0.00000	0.00000
translate	0.10000	0.07062	0.08453	0.09794	0.08919	0.07808
scale	0.50000	0.30602	0.28545	0.37930	0.22112	0.40829
shear	0.00000	0.00000	0.00000	0.00000	0.00000	0.00000
perspective	0.00000	0.00000	0.00000	0.00000	0.00000	0.00000
flipud	0.00000	0.00000	0.00000	0.00000	0.00000	0.00000
fliplr	0.50000	0.38251	0.46339	0.53802	0.46144	0.38413
bgr	0.00000	0.00000	0.00000	0.00000	0.00000	0.00000
mosaic	1.00000	1.00000	0.61543	1.00000	0.79011	0.87937
mixup	0.00000	0.00000	0.00000	0.00000	0.00000	0.00000
cutmix	–	–	0.00000	0.00000	0.00000	0.00000
copy_paste	0.00000	0.00000	0.00000	0.00000	0.00000	0.00000
copy_paste_mode	flip	flip	flip	flip	flip	flip
